# Potential Grape-Derived Contributions to Volatile Ester Concentrations in Wine

**DOI:** 10.3390/molecules20057845

**Published:** 2015-04-29

**Authors:** Paul K. Boss, Anthony D. Pearce, Yanjia Zhao, Emily L. Nicholson, Eric G. Dennis, David W. Jeffery

**Affiliations:** 1 CSIRO Agriculture Flagship, PMB 2, Glen Osmond, SA 5064, Australia; E-Mails: emily.nicholson@csiro.au (E.L.N.); eric.dennis@ubc.ca (E.G.D.); 2 School of Agriculture, Food and Wine, Waite Research Institute, The University of Adelaide, PMB 1, Glen Osmond, SA 5064, Australia; E-Mails: a_pearce@post.com (A.D.P.); yanjia.zhao@student.adelaide.edu.au (Y.Z.); david.jeffery@adelaide.edu.au (D.W.J.)

**Keywords:** grape, wine, ester, amino acid, CoA, yeast, fermentation

## Abstract

Grape composition affects wine flavour and aroma not only through varietal compounds, but also by influencing the production of volatile compounds by yeast. C_9_ and C_12_ compounds that potentially influence ethyl ester synthesis during fermentation were studied using a model grape juice medium. It was shown that the addition of free fatty acids, their methyl esters or acyl-carnitine and acyl-amino acid conjugates can increase ethyl ester production in fermentations. The stimulation of ethyl ester production above that of the control was apparent when lower concentrations of the C_9_ compounds were added to the model musts compared to the C_12_ compounds. Four amino acids, which are involved in CoA biosynthesis, were also added to model grape juice medium in the absence of pantothenate to test their ability to influence ethyl and acetate ester production. β-Alanine was the only one shown to increase the production of ethyl esters, free fatty acids and acetate esters. The addition of 1 mg∙L^−1^ β-alanine was enough to stimulate production of these compounds and addition of up to 100 mg∙L^−1^ β-alanine had no greater effect. The endogenous concentrations of β-alanine in fifty Cabernet Sauvignon grape samples exceeded the 1 mg∙L^−1^ required for the stimulatory effect on ethyl and acetate ester production observed in this study.

## 1. Introduction

Wine is a complex solution containing abundant volatile compounds which contribute to wine aroma and flavour, and consequently impact wine quality and appreciation. Wine aromas are broadly categorised into three groups that reflect their source. Primary aromas are grape-derived volatiles that pass through fermentation often unchanged, and are largely responsible for “varietal” aromas. Secondary aromas, which are by far the greatest pool of volatile molecules, are produced through the winemaking process, with the great majority produced by yeast during alcoholic fermentation as metabolism byproducts [1,2]. Tertiary aromas develop in finished wine through storage and maturation, and result from intermolecular chemical interactions and equilibrium effects as the wine matrix changes.

The volatile composition of most wines, or styles of wine, is very similar, and the varietal differences that exist between wines made from different varieties of grapes are largely due to the relative ratios of the volatile compounds contained within [3]. The types of volatile compounds are diverse, and include esters, higher alcohols, aldehydes, ketones, lactones, acids, phenols, *N*-heterocycles, isoprenoids and sulfur compounds [1,4]. Of these categories, higher alcohols represent the largest volatile pool in terms of concentration, but esters have the largest number of contributing molecules [5].

In this work the term *esters* encompasses both ethyl and acetate esters, which have a significant effect on wine aroma by contributing fruity and floral characteristics [6,7]. These esters are mainly produced by yeast metabolism through fatty acid acyl- and acetyl-Coenzyme A (CoA) pathways [7,8]. CoA is a critical cofactor for a large number of metabolic pathways and is used to activate intermediates during the biosynthesis of medium chain fatty acids (MCFAs); the acyl-CoA intermediates formed are then esterified with ethanol by esterase and transferase enzymes, forming MCFA ethyl esters. Acetate esters, on the other hand, are produced through the condensation of yeast-derived higher alcohols with acetyl-CoA, again under the control of ester-forming enzymes [7,8].

Ester production mainly occurs during fermentation when yeasts generate ample amounts of ethanol and higher alcohols via sugar and amino acid metabolism, respectively [9], so CoA may play a significant role in determining the ester content in wine [10]. For instance, it has been suggested that factors that regulate the production or consumption of acetyl-CoA will in turn alter the amount of esters produced by yeast, and there is evidence for the association of some specific amino acids with CoA biosynthesis [11,12,13]. However, there are no reports directly studying the effect of CoA-related amino acid composition (arising from the grapes) on CoA biosynthesis, and subsequently how a change in CoA concentration might influence ester levels in wine.

Despite the role of acyl transferase enzymes in ester formation by yeast, Saerens *et al.* [14] indicated that the level of gene expression is not the limiting factor for ester production, and that the availability of MCFA precursors has an important role. Moreover, grape chemical composition has been highlighted as being highly influential to the level of production of a suite of aroma compounds, and many grape components are known to be depleted and converted to alcohols and esters through fermentation [15]. It has been widely demonstrated that yeast can liberate volatile molecules from various grape-derived conjugates, such as glycosidically bound and cysteine- and glutathione-conjugated volatile compounds [16,17].

More recent investigations have sought to demonstrate the relationship between grape-derived precursors and wine volatile aromas, such as a study involving ferments in model grape juice medium spiked with varying amounts of natural grape juice to test the impact on wine aromas [18]. This experiment revealed an array of complex interactions across the grape juice proportions added; however, several fermentation esters were identified as having positive, largely linear correlations with grape juice content in the ferments. Following this work, grape-derived aliphatic alcohols and aldehydes were identified as precursors to acetate esters in wine [19]. In particular, the C_6_ compounds (*E*)-2-hexenal, hexanal, (*E*)-2-hexen-1-ol, and hexan-1-ol were shown to be precursors to hexyl acetate, whereas octan-1-ol and benzyl alcohol were identified as precursors to octyl acetate and benzyl acetate, respectively [19]. These examples may also imply the presence of grape-derived MCFAs, or their precursors, contributing to the pool of MFCA ethyl esters liberated during fermentation, which could provide another important link between grape composition and wine volatile profile.

Ultimately, a deeper understanding of the sources and biosynthesis pathways of aroma compounds involves linking viticultural practises, grape variety, grape chemical composition, yeast strain selection, winemaking techniques and wine matrix interactions between aroma constituents with defined sensory outcomes in the finished wine [20,21]. The aim of this study was to address one component of this overall goal, and that was to identify links between potential grape components and wine ester concentrations. A series of model fermentations were conducted with MCFAs and their putative grape-derived precursors, as well as amino acids related to the biosynthesis of CoA, and esters arising in these fermentations were quantified with GC-MS analysis.

## 2. Results and Discussion

### 2.1. Potential Medium Chain Fatty Acid (MCFA)-Derived Precursors of Ethyl Esters

The first part of this study investigated potential grape-derived precursors of the ethyl esters of C_9_ and C_12_ fatty acids (synthesised or commercially available). These two carbon lengths were chosen for two reasons. First, odd-numbered fatty acids and their ethyl esters are found in low abundance in wine, meaning that it is easier to follow the effect of the addition of odd-numbered potential precursors on ethyl ester production as the endogenous concentrations are low. Second, ethyl dodecanoate is extracted at moderate amounts by the SPME method employed in this study compared to shorter even-numbered ethyl esters and hence does not saturate the detector [19]. This allowed the effects of C_12_ precursor addition to be readily detected and allowed the comparison of odd- and even-numbered precursors to be tested in case they are metabolised differently during fermentation.

The potential grape-derived precursors tested included the C_9_ and C_12_ free fatty acids, as well as fatty acids in various conjugated forms. Methyl esters were included because they have been identified in grapes and represent possible contributors to the precursor pool [22]. Carnitine-fatty acid conjugates are potential ethyl ester precursors, as they are the intracellular transport molecule involved in the movement of fatty acids across membranes as part of yeast metabolic activities [23]. Amino acid-fatty acid conjugates have been reported in mammalian systems and in insects (e.g., [24,25]), but as yet have not been described in plants. Nevertheless, fatty acid-amino acid conjugates can act as inducers of plant defence responses when they exist in herbivorous insect saliva (e.g., [26]) and so amino acid conjugates were included as putative precursors.

#### 2.1.1. Free Fatty Acids as Direct Precursors

**Figure 1:**
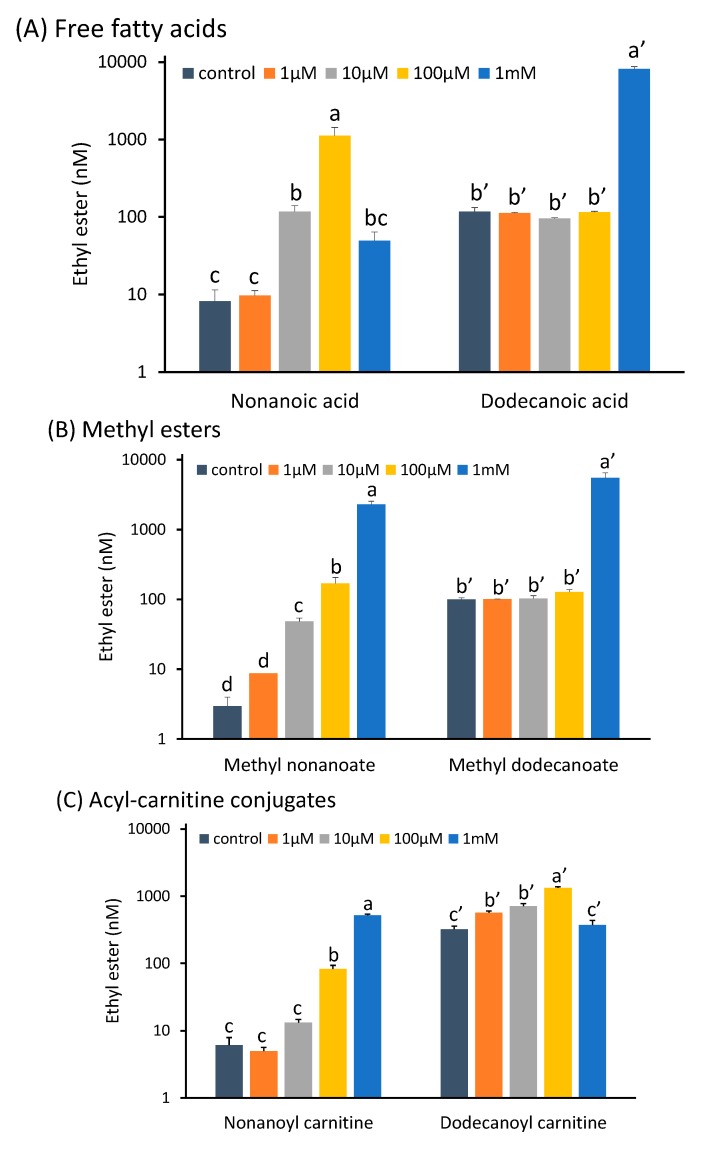
(**A**) Ethyl nonanoate and ethyl dodecanoate concentrations in model wines produced after the addition of increasing amounts of nonanoic acid or decanoic acid to the MGJM; (**B**) Ethyl nonanoate and ethyl dodecanoate concentrations in model wines produced after the addition of increasing amounts of methyl nonanoate or methyl decanoate to the MGJM; (**C**) Ethyl nonanoate and ethyl dodecanoate concentrations in model wines produced after the addition of increasing amounts of nonanoyl carnitine or dodecanoyl carnitine to the MGJM. Histograms represent the mean values (n = 3) and error bars show standard errors. Different letters (e.g., a, b, c, d or aʹ, bʹ, cʹ) denote significant differences between treatments at *p* < 0.05 using ANOVA followed by the Games-Howell *post hoc* test. Note that the y-axis is in log scale.

C_9_ and C_12_ fatty acids were added to model grape juice media (MGJM) fermentations across a range intended to capture the concentrations reported in the literature for comparable molecules in grapes [27,28]. No significant difference in the production of ethyl nonanoate was seen between the control and the fermentations supplemented with 1 μM nonanoic acid (Figure 1A). However, there were significant increases when 10 μM or 100 μM nonanoic acid was added to the MGJM, with ester concentrations reaching 1120 nmol∙L^−1^. The addition of 1 mM nonanoic acid appeared to be toxic to the yeast, as negligible mass was lost throughout the fermentation period. It has been shown in previous studies that free fatty acids can be toxic to yeast at high concentrations [29,30].

No significant increase in the production of ethyl dodecanoate was observed after additions of up to 100 μM dodecanoic acid to the fermentations (Figure 1A). However, there was a large response when 1 mM dodecanoic acid was added, leading to a mean ethyl dodecanoate concentration of 8155 nM (*i.e.*, 1.86 mg∙L^−1^, a sizeable quantity) compared to a mean of 118 nM in the control wine. An increased lag phase was seen in the 1 mM samples, otherwise the fermentation kinetics appeared not to have been impacted in the dodecanoic acid series of additions (data not shown). The esterification of the fatty acid may be a detoxifying action that occurs above a particular toxicity threshold. The findings of Stevens and Hofmeyr [31] support this, as esterification of C_8_ and C_10_ fatty acids reduced their toxicity to the yeast. The results suggest that a concentration threshold for this toxicity exists, and that grape-derived MCFAs could contribute to these concentrations. It would appear that the threshold is much lower for nonanoic acid than for dodecanoic acid and, since most previous research has focused on the effects of even chain MCFAs [8,32], the results of this study suggest a potential odd/even carbon number effect that is worthy of further investigation. Alternatively, the stimulation of ethyl nonanoate production by a lower concentration of nonanoic acid than that observed for dodecanoic acid and its corresponding ethyl ester may reflect the size of the endogenous pool of substrate. As odd-numbered MCFAs are less common than the even-numbered ones, a lower concentration would be required to increase the fatty acid substrate concentration to a point where the production of the ethyl ester is significantly greater than that in the control wine. This may also be the case if the fatty acids have differential abilities to diffuse or be actively transported into the yeast where esterification occurs.

#### 2.1.2. Methyl Esters of MCFAs as Transesterification Substrates

The same concentrations of the methyl esters of nonanoic and dodecanoic acids as used for the free fatty acid experiments were added to MGJM to test the ability of these compounds to influence ethyl ester production. Methyl nonanoate stimulated ethyl nonanoate production when added at concentrations of 10 µM and greater (Figure 1B). The addition of 1 mM methyl nonanoate to the model must resulted in ethyl nonanoate production of 2304 nM (429 ng∙L^−1^) and did not have the toxic effect observed when the free fatty acid was added to the must at this concentration. The addition of methyl dodecanoate produced a similar response to that of the dodecanoic acid additions, with no significant increase in ethyl dodecanoate at any concentration below 1 mM, but a significant increase of ethyl dodecanoate production at that level of addition (Figure 1B). These observations could again be related to either a toxicity response of the yeast above a certain threshold, a reflection of the size of the endogenous substrate pools for these compounds in relation to the amount added to the must, or a consequence of differential rates of transport into the yeast. The metabolism of methyl esters of fatty acids by yeast is seemingly unknown; however, methyl esters of a number of MCFAs have been found in grapes [33], and have been reported in model wines (e.g., [18]). Yeast-mediated transesterification, or alcoholysis, is a probable route of formation of ethyl esters from methyl esters, although the concentrations of these compounds in the fruit are likely to be too low to greatly influence ethyl ester concentrations in wine based on the observations in this experiment.

#### 2.1.3. Acyl-Carnitine Conjugates, Putative Transport Forms of MCFAs

No significant difference was observed in the ethyl nonanoate concentrations of the control wines and those produced after the addition of 1 and 10 μM nonanoyl carnitine. However, the addition of 100 μM nonanoyl carnitine to MGJM significantly increased the ethyl nonanoate concentration in the resulting wine (Figure 1C). Increasing the concentration of nonanoyl carnitine from 100 μM to 1 mM also caused significant increases in ethyl nonanoate production in the subsequent fermentations (Figure 1C). The addition of 1 and 10 μM dodecanoyl carnitine to the MGJM caused a significant difference in ethyl dodecanoate content of the model wines, and this was further enhanced when the model must was supplemented with 100 μM dodecanoyl carnitine (Figure 1C). When 1 mM dodecanoyl carnitine was added to the MGJM the yeast appeared to lose viability and fermentation of the MGJM did not occur, denoted by no loss of weight and no flocculence in the must (data not shown).

The apparent toxicity of the high concentration of dodecanoyl carnitine compared to the free fatty acid was unexpected. Fatty acids appear to be imported into yeast cells in a process that requires activation of the molecule with CoA [34]. The carnitine conjugate may be transported more readily as it is more soluble than the free fatty acid and because it is already activated. The increased transportation of the carnitine conjugate into the cell, followed by liberation of the carnitine, may lead to toxic intracellular levels of the free fatty acid that is not seen when the free fatty acid alone is added to the model must. The mechanism for the transport of extracellular carnitine or conjugates into the cell in *Saccharomyces cerevisiae* is still unclear.

Paradoxically, the nonanoyl carnitine conjugate did not inhibit fermentation when added to the must at a concentration of 1 mM, whereas the free fatty acid did. This may reflect the difference in the fatty acid chain length which may allow more diffusion of the C_9_ fatty acid into the cell compared to both nonanoyl carnitine and free dodecanoic acid. Certainly the stimulation of ethyl nonanoate production was 10-fold lower after the addition of 10 or 100 µM nonanoyl carnitine compared to the equivalent amounts of nonanoic acid (Figure 1). Alternatively, the odd number of carbons in the nonanoyl carnitine conjugate may inhibit any further metabolism of this compound into something that may be toxic to the yeast (for example, the free acid) compared to the even-numbered dodecanoyl carnitine.

The concentration of acyl carnitine conjugates in grape berries is unknown. A study that quantified free carnitine and carnitine esters in many different foods found that fleshy fruits such as apples, guavas and oranges contain concentrations of total carnitine esters of between 0.05–1.45 mg∙kg^−1^ [35]. The grape seed, being a source of lipids, may contain more carnitine esters than the rest of the berry, but further work is required to determine if these conjugates from grapes can alter ester composition in wines.

#### 2.1.4. Acyl-Amino Acid Conjugates Potentially Exploiting Permeases

**Figure 2:**
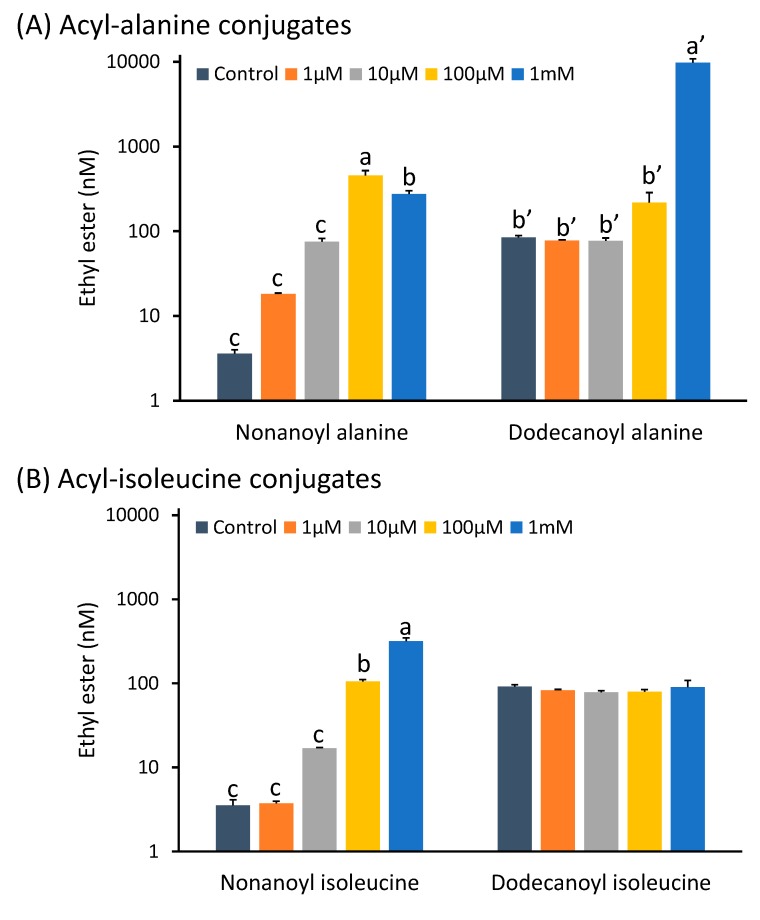
(**A**) Ethyl nonanoate and ethyl dodecanoate concentrations in model wines produced after the addition of increasing amounts of acyl-alanine conjugates to the MGJM. (**B**) Ethyl nonanoate and ethyl dodecanoate concentrations in model wines produced after the addition of increasing amounts of acyl-isoleucine conjugates to the MGJM. Histograms represent the mean values (n = 3) and error bars show standard errors. Different letters (e.g., a, b, c or aʹ, bʹ) denote significant differences between treatments at *p* < 0.05 using ANOVA followed by the Games-Howell *post hoc* test. Note that the y-axis is in log scale.

Addition of nonanoyl alanine to the MGJM at a concentration of 100 μM resulted in a significant increase in the production of ethyl nonanoate in the resulting wine (Figure 2A). Similar to when the free fatty acid was added to the musts, 1 mM nonanoyl alanine appeared to be toxic to the yeast as there was negligible weight loss in these samples; however, ethyl nonanoate concentrations were significantly higher than the control. Nonanoyl isoleucine addition also caused an increase in ethyl nonanoate production when added in concentrations at 100 μM and above (Figure 2B). The toxicity seen when 1 mM nonanoyl alanine was added to the MGJM was not observed after the addition of nonanoyl isoleucine to the MGJM (Figure 2A,B).

Ethyl dodecanoate concentrations were significantly higher only in the wine produced after the addition of 1 mM dodecanoyl alanine to the must, which led to a mean wine concentration of almost 10,000 nmol∙L^−1^, or 2.24 mg∙L^−1^ (Figure 2A). On the contrary, no significant difference was seen in ethyl dodecanoate production at any level of dodecanoyl isoleucine addition (Figure 2B).

The transport mechanism for extracellular alanine and isoleucine is via the general yeast amino acid permeases or other permeases with a less broad specificity [36]. However, the conjugated MCFAs are perhaps more likely to enter the cell in a manner similar to MCFAs. It would appear that the alanine conjugates are transported more easily into the cell as the addition of both the nonanoyl and dodecanoyl variants to the musts resulted in wines with greater concentrations of their respective ethyl esters than did the equivalent addition of the isoleucine conjugates (Figure 2). Alternatively, the alanine conjugates could be more easily esterified or hydrolysed into the free fatty acid, which would increase the available substrate for esterification. Increased transport or hydrolysis of the conjugate could also explain the increased toxicity of the nonanoyl alanine conjugate at 1 mM compared to the isoleucine conjugate. The identification and quantification of acyl-amino acid conjugates in plants has not been reported although it is only recently that such compounds have been described in mammals and insects [24,25]. Future targeted studies may reveal their presence in the plant kingdom as well.

### 2.2. Amino Acids Involved in CoA Biosynthesis

**Figure 3:**
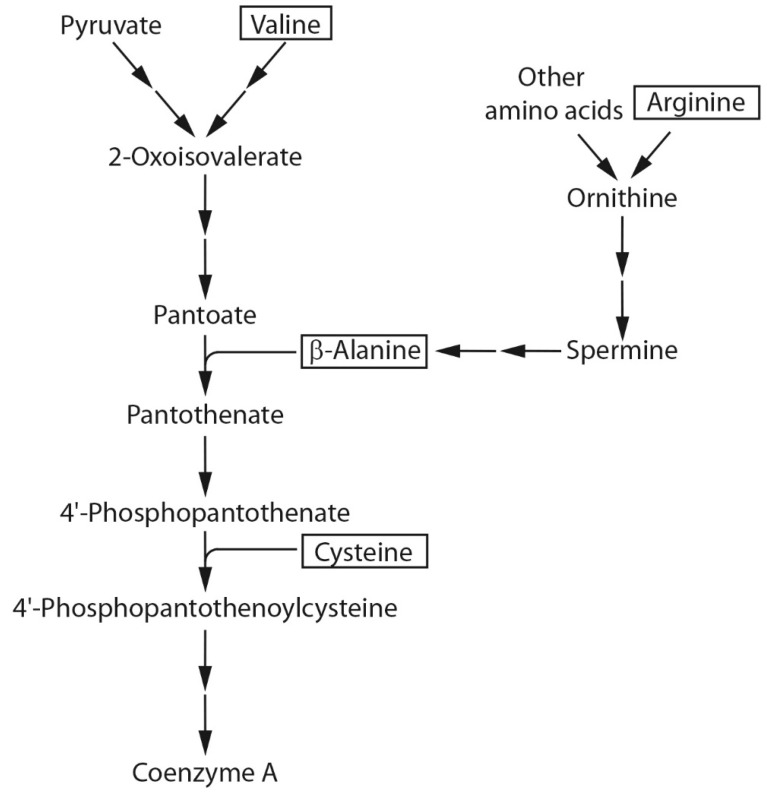
Simplified pathway of CoA biosynthesis in the yeast *S*. *cerevisiae* to emphasise the potential role of the four amino acids (boxed) used in this study.

Four amino acids known to be involved in CoA synthesis (Figure 3) were selected for studies to determine their effect on ester production in MGJM. Valine is associated with CoA generation as it is converted to 2-oxoisovalerate, a precursor of pantothenate [12]. Arginine is the precursor for the biosynthetic production of spermine [13], from which β-alanine is synthesised by *S. cerevisiae* via polyamine degradation. β-Alanine then acts as the intermediate for pantothenic acid biosynthesis [13]. The later steps of CoA synthesis require the reaction of pantothenate with cysteine and ATP (Figure 3).

Since grape juice contains various chemical compounds including pantothenic acid and other precursors that could affect ester syntheses, it is necessary to eliminate these factors when studying the role of these four amino acids in ester production during fermentation. Using a chemically defined MGJM containing a simple mixture of sugars and nutrients (excluding nitrogen sources) can imitate winemaking conditions and is more suitable when gauging the effects of changing one or more variables. However, supplementing model musts with certain amino acids at different levels will also contribute to differences in total nitrogen contents, which can influence ester synthesis during fermentation (e.g., [37]). Hence, total yeast assimilable nitrogen (YAN) was normalised across the experiments by the addition of inorganic ammonium in the form of ammonium chloride (NH_4_Cl). It was also necessary to eliminate pantothenic acid from the normal MGJM as it is through this pathway that we hypothesised that the amino acids may have an effect on ester production.

#### 2.2.1. Stimulation of Ester Production by β-Alanine.

A set of model fermentations were conducted containing one CoA-related amino acid at two concentrations (50 and 100 mg∙L^−1^ β-alanine; 10 and 30 mg∙L^−1^ l-cysteine; 330 and 650 mg∙L^−1^ l-arginine; 30 and 120 mg∙L^−1^ l-valine). The cysteine, arginine and valine concentrations fall within, or are slightly higher than, the typical concentrations of these amino acids found in grape juices [38]. The β-alanine concentrations used were slightly higher than those previously reported for *V. vinifera* grapes [39]. Negative control fermentations did not contain amino acids, and positive control fermentations included all four amino acids at the lowest level added in the individual treatments described above (*i.e*., 50 mg∙L^−1^ β-alanine; 10 mg∙L^−1^ l-cysteine; 330 mg∙L^−1^ l-arginine and 30 mg∙L^−1^ l-valine).

The different treatments affected the length of the fermentation lag phase. The model must supplemented with the mixed amino acids had a lag phase of approximately two days, whereas all the other fermentations had a lag phase of four days, despite them having the same total YAN (data not shown). This could be due to the differences in structural complexity of the nitrogen source [40].

It was hypothesised that the four amino acids of interest would influence ester production through their role in CoA biosynthesis (Figure 3). Therefore, the concentrations of these amino acids could theoretically influence the level of CoA produced by yeast during fermentations, and CoA bearing acetyl/acyl groups would impact on the synthesis of ethyl/acetate esters.

**Figure 4:**
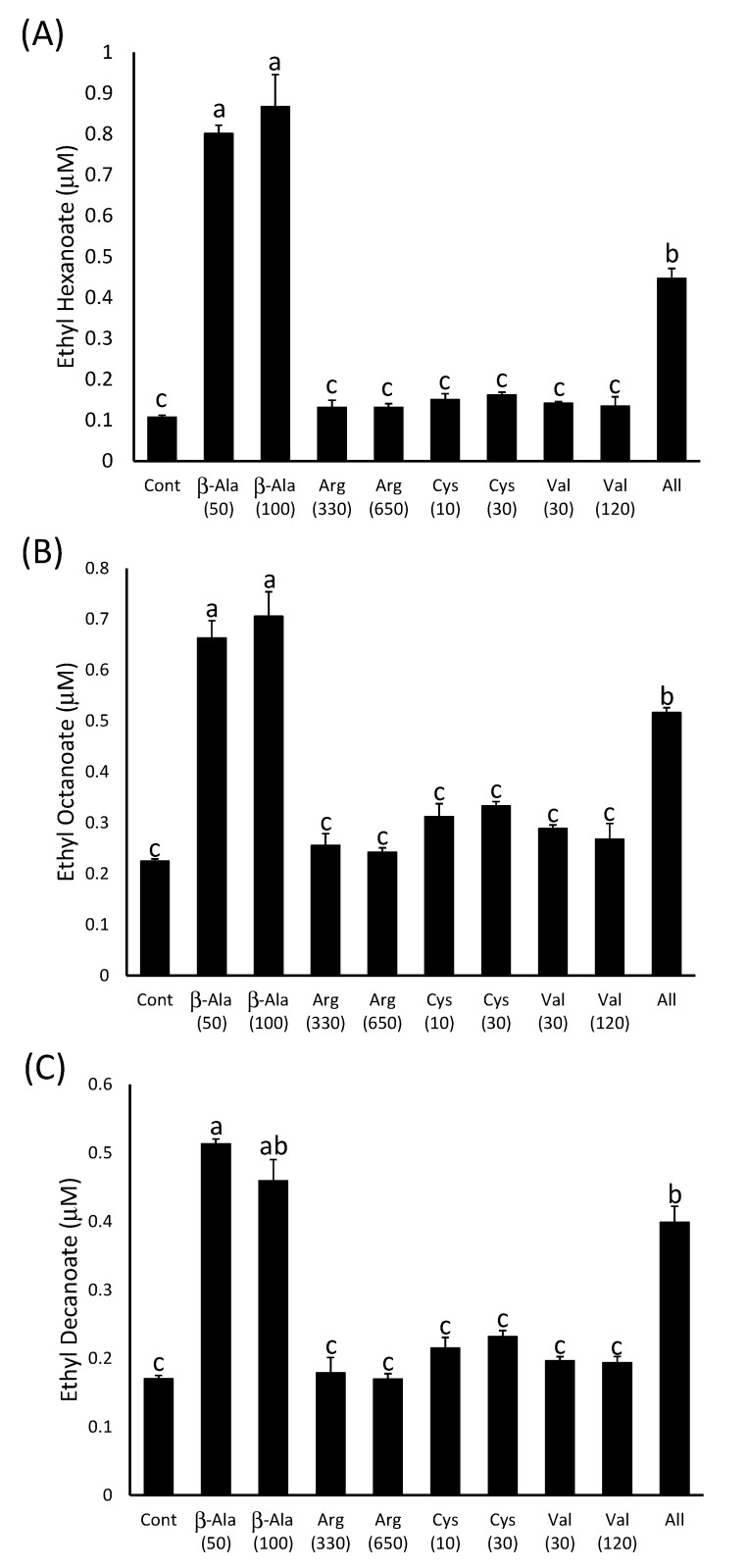
The concentration of (**A**) ethyl hexanoate, (**B**) ethyl octanoate and (**C**) ethyl decanoate in model wines produced after the addition of amino acids as indicated on the x-axis. The number in brackets represents the concentration added in mg∙L^−1^. Histograms represent the mean values (n = 3) and error bars show standard errors. Different letters (e.g., a, b, c) denote significant differences between treatments at *p* < 0.05 using ANOVA followed by Tukey’s *post hoc* test.

The β-alanine-supplemented wines had significantly more ethyl hexanoate, ethyl octanoate and ethyl decanoate (115–125 µg∙L^−1^; 114–122 µg∙L^−1^; 92–103 µg∙L^−1^, respectively) than the control wine (16 µg∙L^−1^; 39 µg∙L^−1^; 34 µg∙L^−1^) or those produced after supplementation of the must with the other three amino acids (Figure 4). However, there were no significant differences between the ethyl ester concentrations in those wines produced from must with either 50 mg∙L^−1^ or 100 mg∙L^−1^ β-alanine added. This suggests that the effect of the addition of this compound on ethyl ester production may be already saturated at 50 mg∙L^−1^. The wines that were produced after the addition of all four amino acids to the must also contained significantly more ethyl esters than the control, but in all three cases the concentrations in the wines were less than those observed in those produced after the addition of 50 mg∙L^−1^ β-alanine alone (Figure 4). This may be due to the difference in the time it took for the fermentations to reach dryness, as this happened approximately two days earlier in the musts with all four amino acids added compared to the other treatments. Volatilisation or metabolism of the ethyl esters may have occurred in these wines while the other treatments were still reaching dryness. Individual additions of arginine, cysteine and valine did not have an impact on ester concentrations as they were not significantly different from the negative control in all cases. This suggests that the yeast is not limited in its ability to produce ethyl esters during fermentation by the supply of arginine, cysteine or valine.

**Figure 5:**
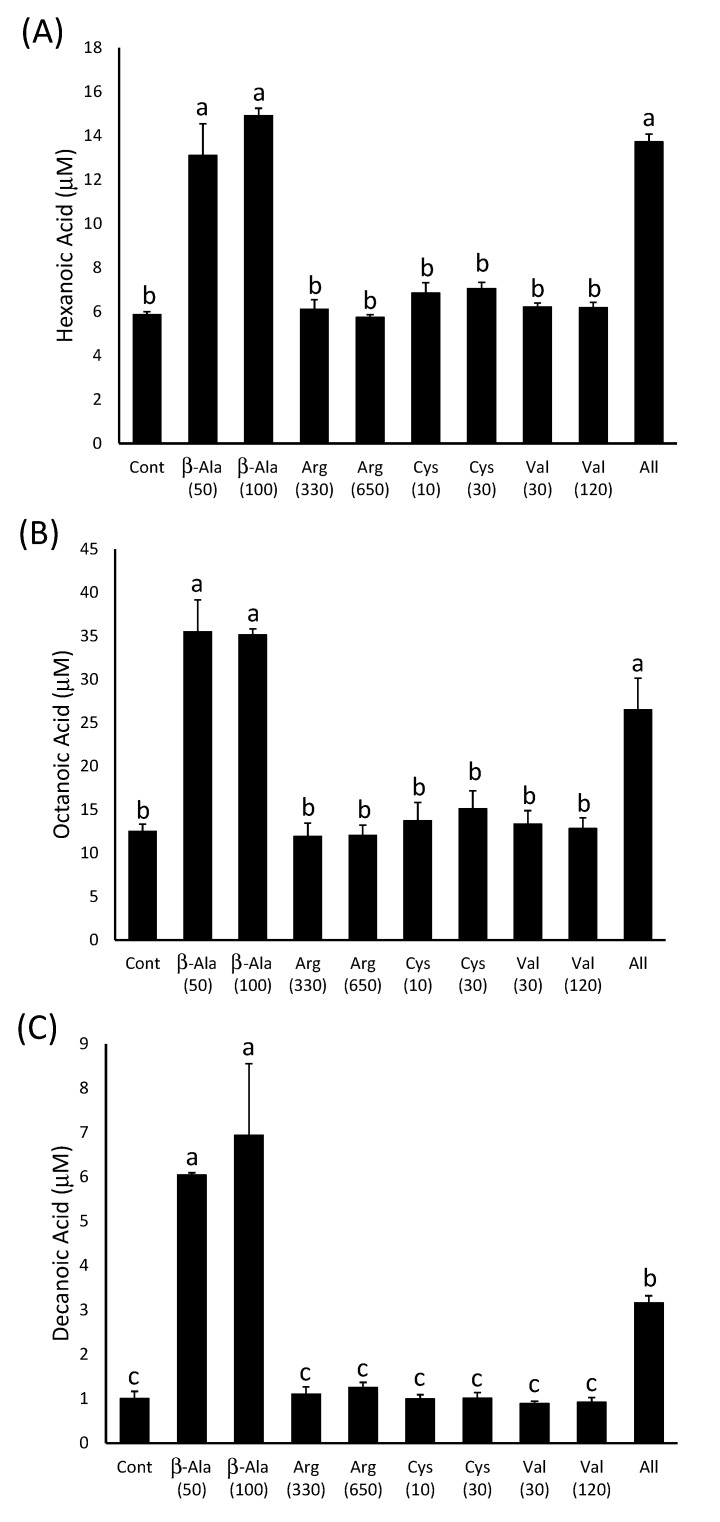
The concentration of (**A**) hexanoic acid, (**B**) octanoic acid and (**C**) decanoic acid in model wines produced after the addition of amino acids as indicated on the x-axis. The number in brackets represents the concentration added in mg∙L^−1^. Histograms represent the mean values (n = 3) and error bars show standard errors. Different letters (e.g., a, b, c) denote significant differences between treatments at *p* < 0.05 using ANOVA followed by Tukey’s *post hoc* test.

Significantly increased concentrations of MCFAs (hexanoic acid 1522–1732 µg∙L^−1^ *vs.* 681 µg∙L^−1^; octanoic acid 3826–5119 µg∙L^−1^ *vs.* 1804 µg∙L^−1^; decanoic acid 545–1195 µg∙L^−1^ *vs.* 174 µg∙L^−1^) were observed in fermentations spiked with 50 and 100 mg∙L^−1^ β-alanine or with all four amino acids compared to the control wine (Figure 5). Therefore, the increase in ethyl ester production may be linked to an increase in the production of MCFAs rather than an increase in activation of existing free fatty acids via an increase in CoA concentration. The lack of any significant difference in hexanoic and octanoic acid content between the wines produced after the addition of four amino acids and those made after β-alanine addition alone may reflect the lower volatility of the MCFAs, compared to the ethyl esters, which reduces their loss from the wine in the two-day time difference between the completions of the fermentations. However, this was not the case for decanoic acid, which was found in significantly lower concentrations when all four amino acids were added compared to those spiked with β-alanine alone (Figure 5C).

**Figure 6:**
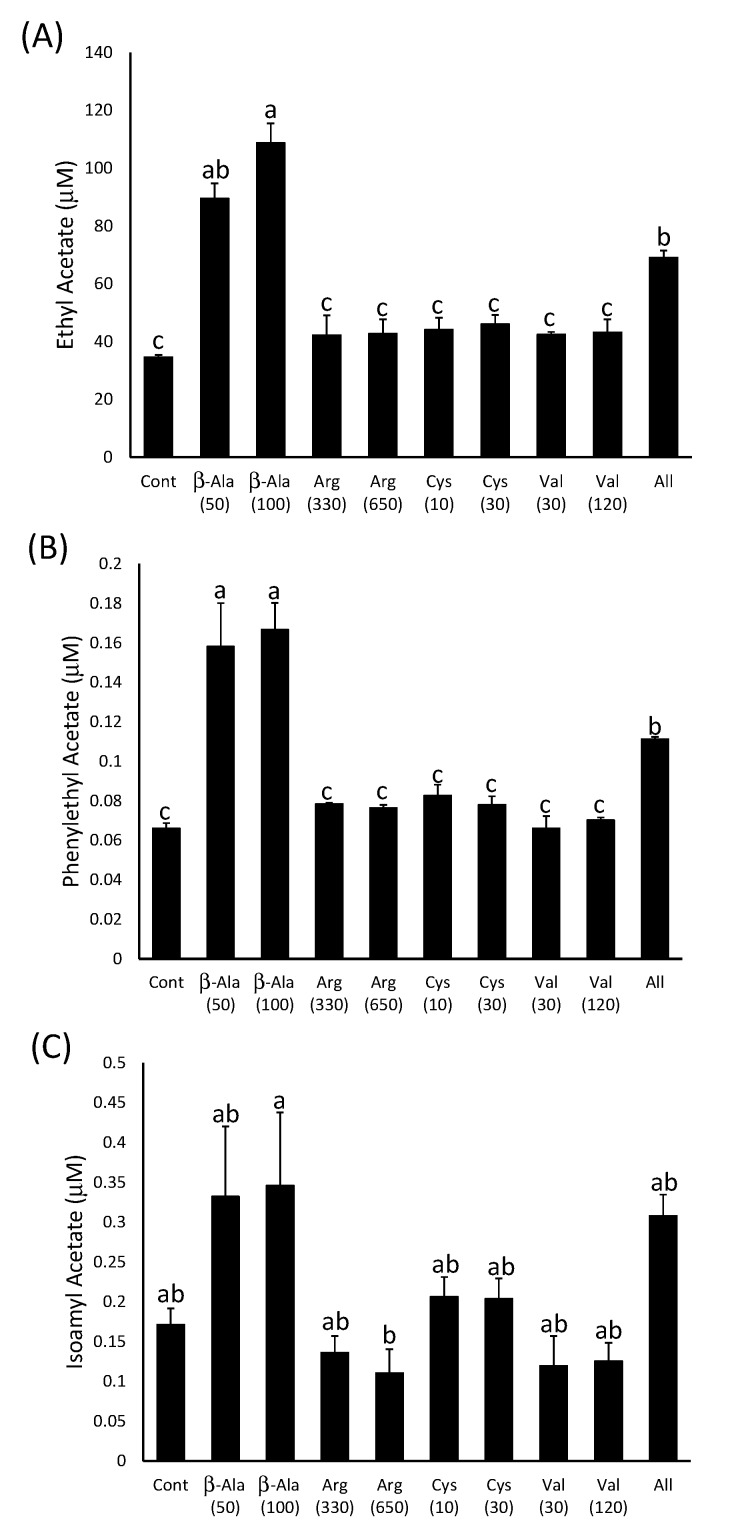
The concentration of (**A**) ethyl acetate, (**B**) isoamyl acetate and (**C**) phenylethyl acetate in model wines produced after the addition of amino acids as indicated on the x-axis. The number in brackets represents the concentration added in mg∙L^−1^. Histograms represent the mean values (n = 3) and error bars show standard errors. Different letters (e.g., a, b, c) denote significant differences between treatments at *p* < 0.05 using ANOVA followed by Tukey’s *post hoc* test.

The concentration of three abundant acetate esters was also determined in the model wines produced after amino acid additions to the model must (Figure 6). Both ethyl acetate and phenylethyl acetate production was higher in the wines produced after β-alanine addition to the musts, either alone or in combination with the other three amino acids, compared to control wine. However, isoamyl acetate concentrations were not significantly higher in the wines produced after β-alanine addition compared to the control. This suggests that isoamyl alcohol concentrations may be limiting production of the corresponding acetate ester, but the concentration of the alcohol moiety of the acetate ester is not limiting for ethyl acetate and phenylethyl acetate production. Presumably, increased CoA synthesis caused by the presence of β-alanine in the must can then increase production of these acetate esters during fermentation.

#### 2.2.2. β-Alanine Concentrations in Grapes are Sufficient to Stimulate Ester Production

The previous experiment demonstrated that additions of 50 and 100 mg∙L^−1^ β-alanine could increase ester concentrations in model fermentations, but cysteine, arginine and valine did not have a significant effect. The concentrations of β-alanine added to the must were above the maximum reported in the literature for *V. vinifera* (33 mg∙L^−1^; [39]) or *Vitis labruscana* (13 mg∙L^−1^; [41]). A collection of 50 Cabernet Sauvignon grape parcels obtained from eight growing regions across two vintages was analysed for β-alanine content to confirm that similar concentrations would be found in Australian-grown fruit. The concentration of β-alanine in these samples ranged from 14 to 66 mg∙L^−1^ with no apparent pattern based on vintage or growing region. Given that some of these values were below the lowest concentration of β-alanine tested in the experiment above (50 mg∙L^−1^), lower concentrations of β-alanine were assessed for their ability to stimulate ester production.

**Figure 7:**
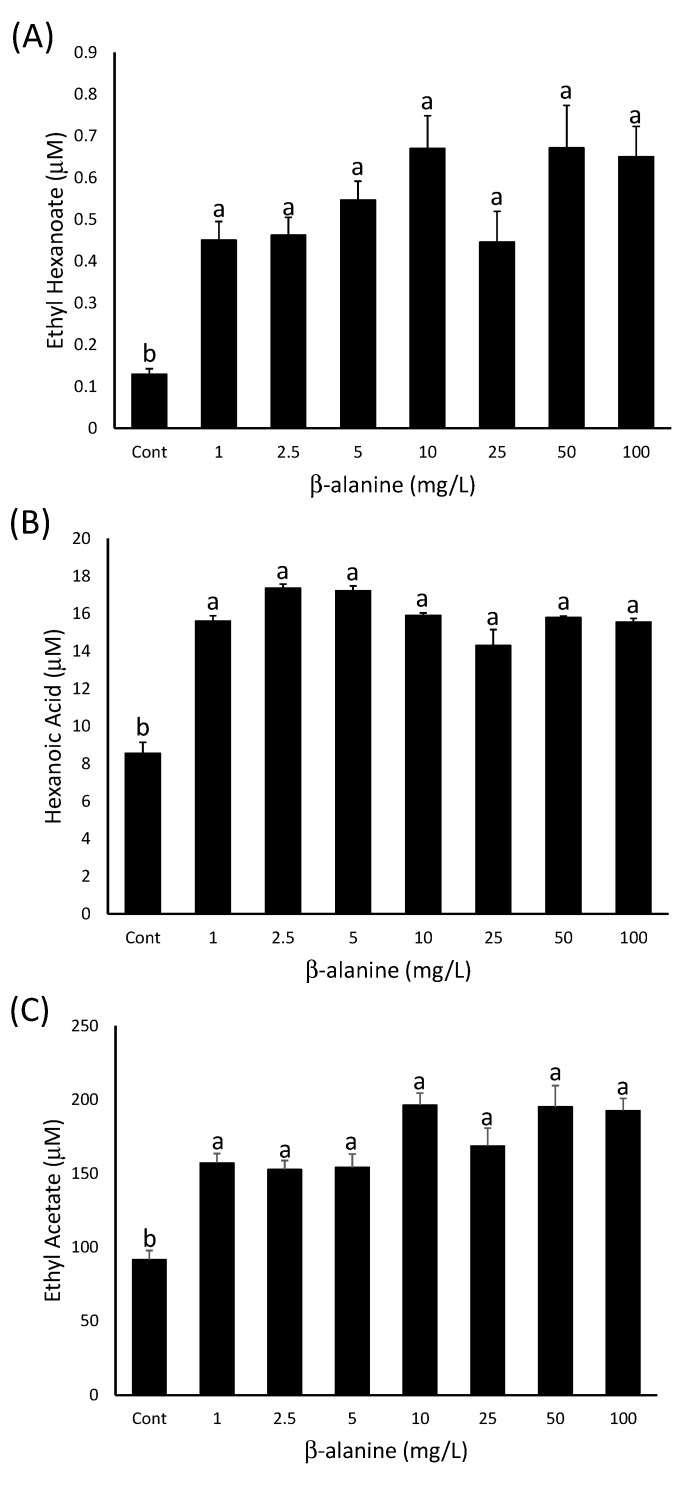
The concentration of (**A**) ethyl hexanoate, (**B**) hexanoic acid and (**C**) ethyl acetate in model wines produced after the addition of increasing amounts of β-alanine as indicated on the x-axis. Histograms represent the mean values (n = 3) and error bars show standard errors. Different letters (e.g., a, b) denote significant differences between treatments at *p* < 0.05 using ANOVA followed by Tukey’s *post hoc* test.

The results indicated that a significant stimulation of ethyl hexanoate production was achieved with the addition of 1 mg∙L^−1^ β-alanine to the model must, and any further increase did not significantly increase ethyl ester production further (Figure 7A). This result was the same for ethyl octanoate and ethyl decanoate production (data not shown). Hexanoic acid, octanoic acid and decanoic acid also accumulated to higher concentrations in the wines produced after the addition of 1 mg∙L^−1^ or more β-alanine—data is shown for hexanoic acid (Figure 7B). Similarly, ethyl acetate was present in higher concentrations in wines produced after any β-alanine addition compared to the control treatment (Figure 7C), although the accumulation of phenylethyl acetate and isoamyl acetate was not significantly different from the control treatments for some of the low β-alanine concentrations (data not shown).

This suggests that 1 mg∙L^−1^ or less of β-alanine is sufficient to stimulate the production of greater amounts of MCFAs and their corresponding ethyl esters, but the addition of more β-alanine does not significantly increase this production further. It is possible that 1 mg∙L^−1^ of β-alanine is enough to account for the requirements of the yeast for maximum fatty acid anabolism and any extra is utilised for other forms of nitrogen metabolism. The effect of β-alanine additions on acetate ester production is probably complicated by the limitation of the alcohol moiety of the ester in the case of isoamyl acetate and phenylethyl acetate; in the case of ethyl acetate, where the alcohol is not limiting, the accumulation is similar to that seen for the ethyl esters (Figure 7).

#### 2.2.3. β-Alanine or Pantothenate Supplementation Have a Similar Effect on Ester Production

To test whether the effect of β-alanine addition to the model must had a similar effect as adding pantothenate, another set of fermentations was prepared with either no additions, addition of 100 mg∙L^−1^ β-alanine (1.12 mM), addition of 267 mg∙L^−1^ panthothenate (1.12 mM), and addition of both β-alanine and pantothenate (same concentration as above).

**Figure 8:**
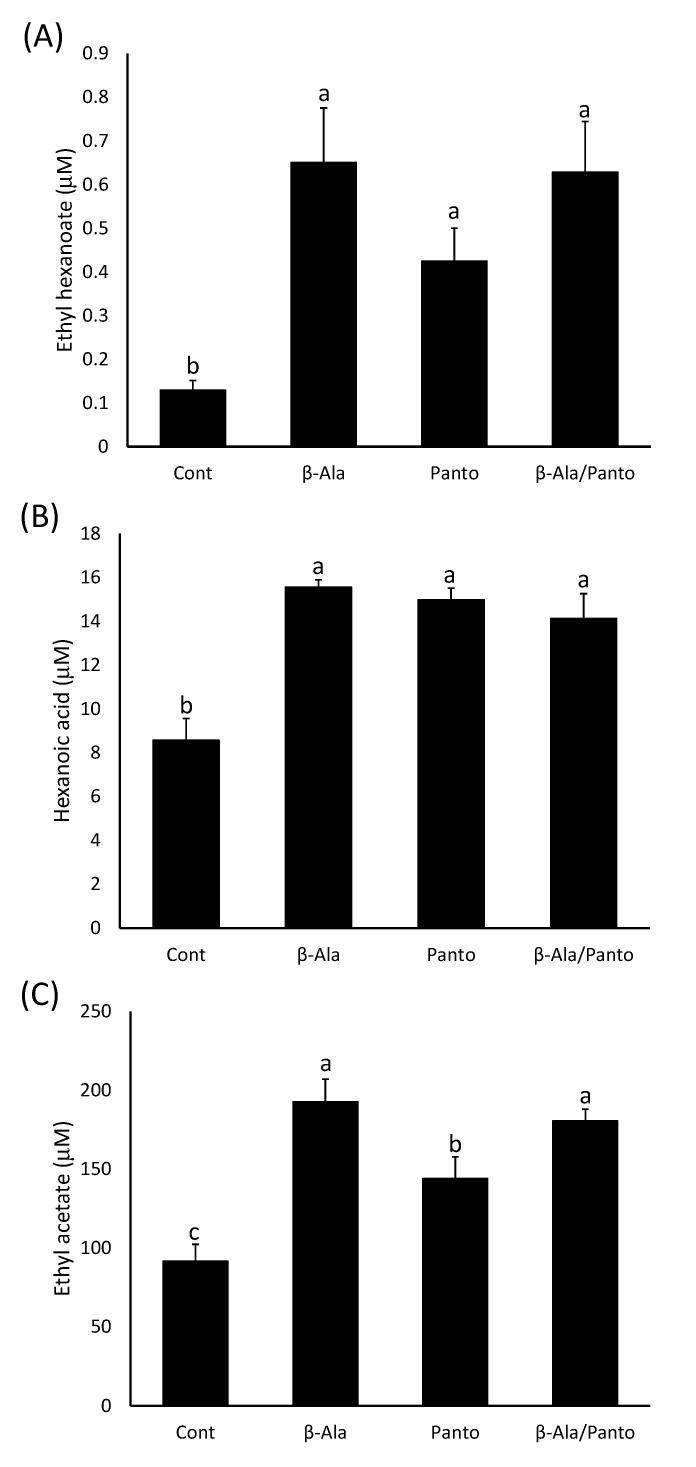
The concentration of (**A**) ethyl hexanoate, (**B**) hexanoic acid and (**C**) ethyl acetate in model wines produced after the addition of β-alanine (β-Ala), pantothenate (Panto) or both compounds (β-Ala/Panto) as indicated on the x-axis. Histograms represent the mean values (n = 3) and error bars show standard errors. Different letters (e.g., a, b, c) denote significant differences between treatments at *p* < 0.05 using ANOVA followed by Tukey’s *post hoc* test.

Ethyl hexanoate production in the fermentations was stimulated to the same degree by the addition of β-alanine, panthothenate or both compounds to the MGJM (Figure 8A). This was also observed for ethyl decanoate, although ethyl octanoate concentrations were not significantly different to the control when only pantothenate was added to the must (data not shown). Hexanoic acid concentrations were significantly greater in the wines produced from all three treatments compared to the control (Figure 8B) and octanoic and decanoic acid responded in the same manner to the supplementation of the must (data not shown). Of the acetate esters, ethyl acetate production was significantly increased in response to the addition of pantothenate to the model must, but even greater production was observed when β-alanine was added (Figure 8C). When isoamyl acetate and phenylethyl acetate were examined, only those wines produced after the addition of β-alanine to the must had greater amounts of these esters compared to the control (data not shown).

In general, both β-alanine and panthothenate could stimulate ester production in the model fermentation system used in these experiments. However, in some cases, β-alanine was more efficient than pantothenate at increasing ester concentrations. This observation seems counterintuitive given that pantothenate is produced from pantoate and β-alanine during CoA biosynthesis (Figure 3). However, there may be differences in the ability of yeast to transport these compounds into the cell which in turn could influence CoA and ester production. It is known that pantothenate is transported by the Fen2p symporter, whereas β-alanine is transported mainly by the general amino acid permease Gap1p, although other transporters are involved [42]. The redundancy in the β-alanine transport system may make its transport more efficient and hence the concentration in the yeast cytoplasm may be greater than that of pantothenate when both are at the same concentration in the media. Alternatively, β-alanine may be used in other metabolic pathways that can also stimulate ester production independent of CoA biosynthesis.

A survey of panthothenate concentrations in grapes grown in the Pacific northwest of the USA found that they ranged from 0.18 to 1.26 mg∙L^−1^ [43]. This is lower than the values reported for β-alanine (this manuscript, [39]) which would suggest that β-alanine may have a more important role in CoA production during fermentation of grapes than pantothenate.

## 3. Experimental Section

### 3.1. Material

#### 3.1.1. Chemicals

Commercially available compounds used for fermentation spiking were purchased from Sigma-Aldrich (Castle Hill, NSW, Australia) and used without further preparation. Putative precursor compounds were synthesised from commercially available reagents and other chemicals outlined below, such as amino acids, salts and sugars, were analytical reagent grade (Sigma-Aldrich). All solvents used were analytical reagent (Sigma-Alrich) or HPLC grade (Merck, Kilsyth, VIC, Australia) and water was obtained from a Milli-Q purification system (Millipore, North Ryde, NSW, Australia). Solutions were % v/v with the balance made up with Milli-Q water, unless otherwise noted.

#### 3.1.2. Nuclear Magnetic Resonance (NMR)

Proton (^1^H) and carbon (^13^C) NMR spectra were recorded with a Bruker Avance III spectrometer operating at 600 MHz for proton and 150 MHz for carbon nuclei. Chemical shifts were recorded as δ values in parts per million (ppm). Spectra were acquired in methanol-d_4_ at ambient temperature, using the solvent residual peak as internal reference, and resonances were assigned by routine 2D correlation experiments.

#### 3.1.3. High-Resolution Mass Spectrometry (HR-MS)

Spectra were obtained on a Bruker microTOF-Q II with electrospray ionization (ESI) in positive mode. Samples dissolved in 2:1 methanol/water at concentrations of approximately 1−2 mg∙L^−1^ were analysed by flow injection.

#### 3.1.4. HPLC-MS Instrumentation

Analysis of synthesised compounds was undertaken using a ThermoFinnigan Surveyor HPLC connected to a ThermoFinnigan LCQ Deca XP Plus mass spectrometer. Electrospray ionisation in positive ion mode was used and data acquisition and processing were performed using Xcalibur software (version 1.3, Thermo Scientific, San Jose, CA, USA).

#### 3.1.5. GC-MS Instrumentation

Analyses of synthetic standards and fermentation esters were conducted with an Agilent (Palo Alto, CA, USA) 7890A gas chromatograph, equipped with a Gerstel (Mülheim an der Ruhr, Germany) MP2 autosampler fitted with a DVB/CAR/PDMS SPME fibre (2 cm, 50/30 μm, Supelco, Bellefonte, PA, USA), coupled to an Agilent 5975C mass spectrometer as described previously [19]. Selected ion monitoring (SIM) was used for detection of analytes for quantitation and scan mode (*m*/*z* 35−350; scan rate, 4.45 scans/s) was used for synthesised standards. Compounds were identified by comparing their mass spectra to spectral libraries and from the analysis of authentic reference compounds.

#### 3.1.6. Melting Points

A Buchi melting point B-540 unit was used, and melting points were uncorrected.

### 3.2. Synthesis of Compounds

#### 3.2.1. *d*_5_-Ethyl Nonanoate

To a stirred solution of *d*_6_-ethanol (500 μL, 8.64 mmol) in anhydrous CH_2_Cl_2_ (4 mL) under N_2_ at room temperature was added pyridine (614 μL, 7.59 mmol) followed by 4-dimethylaminopyridine (DMAP) (0.106 g, 0.868 mmol). After dissolution, nonanoyl chloride (1.50 mL, 8.32 mmol) was added drop-wise to the solution at 0 °C, causing the formation of a white precipitate. Additional anhydrous CH_2_Cl_2_ (3 mL) was added and the solution was stirred overnight at room temperature, before being quenched with NaHCO_3_ (10 mL). The phases were separated, and the aqueous phase was extracted with CH_2_Cl_2_ (3 × 20 mL), and the combined organic phases were washed with brine (20 mL), dried (NaSO_4_), and filtered before being concentrated *in vacuo* to yield a yellow/brown oil. Purification by column chromatography on silica (85% hexane/EtOAc, R_f_ = 0.45) gave the title compound (1.41 g, 7.37 mmol, 89%) as a colorless oil (97% pure by GC-MS). ^1^H-NMR (600 MHz, CD_3_OD): δ 2.29 (2H, t, *J* = 7.6 Hz, H_2_); 1.62 (2H, app q, *J* = 7.4 Hz, H_3_); 1.35–1.22 (10 H, m, H_4-8_); 0.88 (3H, t, *J* = 7.0 Hz, H_9_); ^13^C-NMR (150 MHz, MeOH-*d*_4_): δ 173.97 (C_1_); 59.40 (*J*_C-D_ = 22 Hz, *C*D_2_); 34.39 (C_2_); 31.79 (C_7_); 29.21 (C_4_); 29.13 (C_5_); 29.11 (C_6_); 24.97 (C_3_); 22.63 (C_8_); 14.09 (C_9_); 13.21 (*J*_C-D_ = 19.4 Hz, *C*D_3_). ESI-HRMS (*m/z*): Calcd for C_11_H_18_D_5_O_2_^+^ ([M+H]^+^), 192.2006; found 192.2022. EI-MS (*m*/*z*) (%) 191 (M^+^, 3), 162 (12), 148 (13), 141 (19), 120 (6), 106 (39), 93 (100), 84 (6), 74 (28), 61 (20), 57 (13), 55 (17).

#### 3.2.2. *d*_5_-Ethyl Dodecanoate

This compound was prepared using the procedure described for *d*_5_-ethyl nonanoate, using dodecanoyl chloride (1.85 mL, 8.29 mmol), *d*_6_-ethanol (500 μL, 8.64 mmol), pyridine (614 μL, 7.6 mmol), and DMAP (105.6 mg, 864 μmol) in CH_2_Cl_2_ (13 mL). Purification by column chromatography on silica (85% hexane/EtOAc, R_f_ = 0.59) gave the title compound (1.85 g, 7.93 mmol, 96%) as a colourless oil, which was >99% pure (GC-MS). ^1^H-NMR (MeOH-*d*_4_) δ 2.29 (2H, t, *J* = 7.6 Hz, H_2_); 1.62 (2H, app q, *J* = 7.2 Hz, H_3_); 1.35–1.22 (16H, m, H_4-8_); 0.88 (3H, t, *J* = 7.0 Hz, H_12_); ^13^C-NMR (MeOH-*d*_4_) δ 173.97 (C_1_); 59.39 (*J*C-D = 22.2Hz, CD_2_); 58.88 (C_13_); 34.38 (C_2_); 31.88 (C_10_); 29.58 (C_4,5_); 29.44 (C_6_); 29.32 (C_7_); 29.25 (C_8_); 29.13 (C_9_); 24.97 (C_3_); 22.67 (C_11_); 14.11 (C_12_); 13.31 (C_14_); 13.2 (*J*C-D = 19.5 Hz, CD_3_). ESI-HRMS (*m/z*): Calcd for C_14_H_24_D_5_O_2_^+^ ([M+H]^+^), 234.2467; found, 234.2476. EI-MS (*m*/*z*) (%) 233 (M^+^, 6), 204 (5), 190 (12), 183 (12), 162 (15), 148 (8), 120 (5), 106 (52), 98 (5), 94 (12), 93 (100), 84 (6), 83 (5), 74 (24), 69 (7), 63 (6), 61 (11), 57 (10), 55 (16), 43 (16), 41 (16), 34 (6), 32 (9), 29 (6), 28 (33).

#### 3.2.3. (2*R/S*)-3-Carboxy-2-(nonanoyloxy)-*N,N,N*-trimethylpropan-1-aminium (*N*-Nonanoyl carnitine)

Carnitine derivatives were prepared based on the method of Bøhmer and Bremer [44]. To a stirred solution of trifluoroacetic acid (TFA) (500 μL) at room temperature containing (±)-carnitine chloride (1.00 g, 5 mmol) was added dropwise nonanoyl chloride (1.77 g, 10 mmol), at which time a white precipitate formed. The reaction was maintained at 50 °C with stirring overnight. After cooling to room temperature, acetone (5.5 mL) was added, stirring was maintained for 1 hour and the precipitate that formed was removed via centrifugation. Diethyl ether (~3 mL) was added dropwise to incipient cloudiness, followed by cooling to 0 °C. Additional Et_2_O (10 mL) was added once crystallisation was well underway. Once crystallisation was complete, centrifugation and resuspension in Et_2_O (40 mL) was used to wash the crystals (repeated two more times), followed by isolation and drying. Purification by recrystallisation in MeOH/Et_2_O produced the known [45] title compound (1.16 g, 3.43 mmol, 69%) as a white crystalline solid, which was >99% pure (HPLC-MS), mp 155.2–157.7 °C. ^1^H-NMR (MeOH-*d*_4_) δ 5.63 (1H, app q, *J* = 6.9 Hz); 4.92 (1H, bs); 3.49 (1H, dd, *J* = 14.4, 8.6 Hz); 3.75 (1H, dd, *J* = 14.4, 0.90 Hz); 3.23 (9H, s); 2.79–2.76 (2H, m); 2.39 (2H, m); 1.63 (2H, q, *J* = 7.35 Hz); 1.38–1.25 (10H, m); 0.90 (3H, t, *J* = 7.1 Hz). ^13^C-NMR (MeOH-*d*_4_) δ 174.27, 172.54, 69.50, 66.35, 54.68, 37.93, 35.22, 33.11, 30.50, 30.39, 30.31, 25.83, 23.84, 14.60.

#### 3.2.4. (2*R/S*)-3-Carboxy-2-(dodecanoyloxy)-*N,N,N*-trimethylpropan-1-aminium (*N*-Dodecanoyl carnitine)

Using the same reaction and isolation procedure as that for *N*-nonanoyl carnitine above, *N*-dodecanoyl carnitine was prepared using (±)-carnitine chloride (1.00 g, 5 mmol) and dodecanoyl chloride (2.19 g, 10 mmol) in (TFA) (500 μL). Purification by recrystallisation in MeOH/Et_2_O produced the known [45] title compound (1.32 g, 3.47 mmol, 69%) as a white crystalline solid, which was 93% pure (HPLC-MS), mp 167.5–175.3 °C. ^1^H-NMR (MeOH-*d*_4_) δ 5.63 (1H, app q, *J* = 6.9 Hz); 3.89 (1H, dd, *J* = 14.3, 8.6 Hz); 3.74 (1H, d, *J* = 14.3 Hz); 3.22 (9H, s); 2.79–2.76 (2H, m); 2.39 (2H, app t, *J* = 7.5 Hz); 1.63 (2H, q, *J* = 7.3 Hz); 1.37–1.24 (16H, m); 0.90 (3H, t, *J* = 7.0 Hz). ^13^C-NMR (MeOH-*d*_4_) δ 174.13, 172.41, 69.37, 66.21, 37.77, 35.08, 33.07, 30.74, 30.59, 30.48, 30.42, 30.18, 25.71, 23.75, 14.48.

#### 3.2.5. Methyl 2-(Nonanoylamino)propanoate (*N*-Nonanoyl Alanine Methyl Ester)

Amide coupling was performed according to the method of Staab [46] and Staab *et al.* [47] using carbonyl diimidazole (CDI). To a stirred solution of nonanoic acid (322.9 μL, 2 mmol) in anhydrous THF (4.39 mL) under N_2_ was added CDI (324.3 mg, 2 mmol), causing effervescence from liberated CO_2_. Stirring was continued at room temperature for 2 h and methyl l-alaninate (279.2 mg, 2 mmol) was added, resulting in separation of a brown oily residue. Stirring at room temperature continued overnight, by which time a white precipitate had formed. The solvent was removed *in vacuo*, resulting in the formation of white crystals in an oily residue, which were then taken up in Et_2_O (10 mL), EtOAc (20 mL), and H_2_SO_4_ (1 M, 15 mL). The phases were separated and the organic phase was washed successively with H_2_SO_4_ (1 M, 3 × 15 mL), water (20 mL), and brine (20 mL). The organic phase was dried (MgSO_4_), filtered, and concentrated *in vacuo*, producing a white crystalline solid, (551.3 mg, 2.27 mmol, 113%) which was used crude in the subsequent reaction. A small portion was purified for characterisation by column chromatography on silica (80% CH_2_Cl_2_/EtOAc, R_f_ = 0.51) to give the title compound as a pale yellow powder, which was 98% pure (HPLC-MS), mp 36.9–38.8 °C. ${\left[\alpha \right]}_{D}^{20}$= +4.8 (*c* 0.620, CHCl_3_). ^1^H-NMR (MeOH-*d*_4_) δ 6.05–6.12 (1H, m, N*H*); 4.61 (1H, app q, *J* = 7.2 Hz, H_11_); 2.20 (2H, t, *J* = 7.6 Hz, H_2_); 1.63 (2H, app q, *J* = 7.4 Hz, H_3_); 1.40 (3H, d, *J* = 7.1 Hz, H_12_); 1.35–1.22 (10H, m, H_4-8_); 0.87 (3H, t, *J* = 7.0 Hz, H_12_); ^13^C-NMR (MeOH-*d*_4_) δ 173.72 (C_10_); 172.65 (C_1_); 52.42 (C_13_); 47.81 (C_11_); 36.54 (C_2_); 31.77 (C_7_); 29.25 (C_4_); 29.17 (C_5_); 29.09 (C_6_); 25.53 (C_3_); 22.60 (C_8_); 18.56 (C_12_); 14.06 (C_9_). ESI-HRMS (*m/z*): Calcd for C_13_H_26_NO_3_^+^ ([M+H]^+^), 244.1985; found, 244.1907.

#### 3.2.6. 2-(Nonanoylamino)propanoic acid (*N*-nonanoyl alanine)

To a stirred solution of THF (24.4 mL) containing *N*-nonanoyl methyl ester (501.3 mg, 1.24 mmol) was added aqueous NaOH (1 M, 9.76 mL) and the solution was stirred at room temperature for 5 hours. After adjustment to pH 3 with HCl (1 M), the solution was extracted with CH_2_Cl_2_ (3 × 20 mL). The combined organic fractions were washed with water (20 mL) and brine (20 mL), before being dried (MgSO_4_), filtered and concentrated *in vacuo* to produce a colourless oil. Purification by crystallisation in MeOH/H_2_O gave the title compound (414.8 mg, 1.81 mmol, 90%) as a colourless oil, which was >98% pure (HPLC-MS). ${\left[\alpha \right]}_{D}^{20}$ = −17.2 (*c* 0.580, CHCl_3_). ^1^H-NMR (MeOH-*d*_4_) δ 8.35 (1H, bs, CO_2_*H*); 6.36–6.28 (1H, m, N*H*); 4.58 (1H, app q, *J* = 7.1 Hz, H_11_); 2.24 (2H, t, *J* = 7.7 Hz, H_2_); 1.63 (2H, app q, *J* = 7.2 Hz, H_3_); 1.46 (3H, d, *J* = 7.2 Hz, H_2_); 1.35–1.22 (10H, m, H_4-8_); 0.88 (3H, t, *J* = 7.0 Hz, H_9_); ^13^C-NMR (MeOH-*d*_4_) δ 175.93 (C_10_); 174.11 (C_1_); 48.21 (C_11_); 36.40 (C_2_); 31.76 (C_7_); 29.22 (C_4_); 29.13 (C_5_); 29.09 (C_6_); 25.54 (C_3_); 22.60 (C_8_); 18.06 (C_12_); 14.06 (C_9_). ESI-HRMS (*m/z*): Calcd for C_12_H_24_NO_3_^+^ ([M+H]^+^), 230.1748; found, 230.1751.

#### 3.2.7. Methyl 2-(Dodecanoylamino)propanoate (*N*-dodecanoyl alanine methyl ester)

Using the same reaction and isolation procedure as that for *N*-nonanoyl alanine methyl ester above, *N*-dodecanoyl alanine methyl ester was prepared using dodecanoic acid (817.6 μL, 4 mmol), CDI (648.6 mg, 4 mmol) and methyl l-alaninate (558.3 mg, 4 mmol) in THF (8.79 mL), producing a white, waxy solid (1.108 g, 3.88 mmol, 97%). Purification by recrystallisation was attempted in CH_2_Cl_2_/hexane and EtOH/hexane, but the product formed an organogel, so the crude product underwent the saponification procedure described below. A small portion was purified for characterisation by column chromatography on silica (80% CH_2_Cl_2_/EtOAc, R_f_ = 0.47) to give the title compound as a white crystalline solid, which was >99% pure (HPLC-MS), mp 58.8–61.2 °C. ${\left[\alpha \right]}_{D}^{20}$ = +5.0 (*c* 0.600, CHCl_3_). ^1^H-NMR (MeOH-*d*_4_) δ 6.10–6.02 (1H, m, N*H*); 4.61 (1H, app q, *J* = 7.2 Hz, H_14_); 3.75 (3H, s, H_16_); 2.20 (2H, t, *J* = 7.7 Hz, H_3_); 1.63 (2H, app q, *J* = 7.4 Hz, H_2_); 1.40 (3H, d, *J* = 7.1 Hz, H_15_); 1.35–1.20 (16H, m, H_4-11_); 0.88 (3H, t, *J* = 7.0 Hz, H_12_); ^13^C-NMR (MeOH-*d*_4_) δ 173.87 (C_13_); 172.83 (C_1_); 52.56 (C_16_); 47.94 (C_14_); 36.67 (C_2_); 31.04 (C_10_); 29.56 (C_4-9_); 25.67 (C_3_); 22.77 (C_11_); 18.70 (C_15_); 14.06 (C_12_). Melting point and spectral data were in complete accord with those previously reported [48].

#### 3.2.8. 2-(Dodecanoylamino)propanoic acid (*N*-dodecanoyl alanine)

Using the same reaction and isolation procedure as that for *N*-nonanoyl alanine above, *N*-dodecanoyl alanine was prepared using *N*-dodecanoyl alanine methyl ester (1.05 g, 3.69 mmol), THF (64.7 mL), and aqueous NaOH (1 M, 25.9 mL) to produce a white powder. Purification by recrystallisation in MeOH/H_2_O gave the title compound (928.8 mg, 3.42 mmol, 86%) as a white powder, which was >98% pure (HPLC-MS), mp 82.8–83.7 °C. ^1^H-NMR (MeOH-*d*_4_) δ 6.30–6.23 (1H, m, N*H*); 4.57 (1H, app q, *J* = 7.1 Hz, H_14_); 2.24 (2H, t, *J* = 7.6 Hz, H_2_); 1.62 (2H, app q, *J* = 7.3 Hz, H_3_); 1.46 (3H, d, *J* = 7.2 Hz, H_15_); 1.35–1.21 (16H, m, H_4-11_); 0.88 (3H, t, *J* = 7.0 Hz, H_12_); ^13^C-NMR (MeOH-*d*_4_) δ 175.62 (C_13_); 174.06 (C_1_); 48.22 (C_14_); 36.41 (C_2_); 31.88 (C_10_); 29.67 (C_4_); 29.57 (C_5_); 29.44 (C_6_); 29.31 (C_7_); 29.27 (C_8_); 29.15 (C_9_); 25.53 (C_3_); 22.66 (C_11_); 18.00 (C_15_); 14.10 (C_12_). Melting point and spectral data were in complete accord with those previously reported [48].

#### 3.2.9. Methyl 2-(Nonanoylamino)-3-methylpentanoate (*N*-nonanoyl isoleucine methyl ester)

Using the same reaction and isolation procedure as that for *N*-nonanoyl alanine methyl ester above, *N*-nonanoyl isoleucine methyl ester was prepared using nonanoic acid (645.9 μL, 4 mmol), CDI (648.6 mg, 4 mmol) and methyl l-isoleucinate (726.6 mg, 4 mmol) in THF (8.79 mL), producing a pale brown oil (1.022 g, 3.58 mmol, 90%). A small portion was purified for characterisation by column chromatography on silica (85% CH_2_Cl_2_/EtOAc, R_f_ = 0.57) producing the title compound as a pale brown oil, which was >98% pure (HPLC-MS). ^1^H-NMR (MeOH-*d*_4_) δ 5.92–5.99 (1H, m, N*H*); 4.62 (1H, dd, *J* = 8.6, 5.0 Hz, H_11_); 2.22 (3H, t, *J* = 7.6 Hz, H_2_); 1.92–1.84 (1H, m, H_12_); 1.64 (2H, app q, *J* = 7.3 Hz, H_3_); 1.43 (1H, dqd, *J* = 14.8, 7.4, 4.7 Hz, H_3_); 1.27 (11H, m, H_4-8_); 1.17 (1H, dqd, *J* = 14.8, 7.4, 7.3 Hz, H_13_); 0.92 (3H, t, *J* = 7.4 Hz, H_14_); 0.90 (3H, d, *J* = 6.8 Hz, H_15_); 0.88 (3H, t, *J* = 7.0 Hz, H_9_). ^13^C-NMR (MeOH-*d*_4_) δ 172.87 (C_1_); 172.73 (C_10_); 56.11 (C_11_); 52.06 (C_16_); 37.97 (C_12_); 36.70 (C_2_); 31.78 (C_7_); 29.26 (C_4_); 29.21 (C_5_); 29.12 (C_6_); 25.64 (C_3_); 25.18 (C_15_); 22.61 (C_8_); 15.38 (C_13_); 14.08 (C_9_); 11.55 (C_14_). ESI-HRMS (*m/z*): Calcd for C_16_H_32_NO_3_^+^ ([M+H]^+^), 286.2368; found, 286.2377.

#### 3.2.10. 2-(Nonanoylamino)-3-methylpentanoic acid (*N*-nonanoyl isoleucine)

Using the same reaction and isolation procedure as that for *N*-nonanoyl alanine above, *N*-nonanoyl isoleucine was prepared using *N*-nonanoyl isoleucine methyl ester (0.972 g, 3.41 mmol), THF (4.25 mL), and aqueous NaOH (1 M, 3.4 mL) to produce a white solid. Recrystallisation in 1:1 EtOAc/hexane produced the title compound (590 mg, 2.17 mmol, 64%) as fine white crystals that were 98% pure (HPLC-MS). ${\left[\alpha \right]}_{D}^{20}$ = +26.9 (*c* 1.135, CHCl_3_). ^1^H-NMR (MeOH-*d*_4_) δ 8.45–7.72 (1H, bs, CO2*H*); 6.24–6.16 (1H, m, N*H*); 4.63 (1H, dd, *J* = 8.5, 4.7 Hz, H_11_); 2.31–2.22 (2H, m, H_2_); 1.99–1.92 (1H, m, H_12_); 1.63 (2H, app q, *J* = 7.4 Hz, H_3_); 1.50 (1H, dqd, *J* = 13.4, 7.4, 4.5 Hz, H_3_); 1.35–1.21 (10H, m, H_4-8_); 1.26–1.17 (1H, m, H_13_); 0.95 (3H, d, *J* = 6.8 Hz, H_15_); 0.94 (3H, t, *J* = 7.4 Hz, H_14_); 0.88 (3H, t, *J* = 7.0 Hz, H_9_). ^13^C-NMR (MeOH-*d*_4_) δ 175.40 (C_1_); 174.03 (C_10_); 56.41 (C_11_); 37.62 (C_12_); 36.62 (C_2_); 31.77 (C_7_); 29.23, 29.17, 29.11 (C_4,5,6_); 25.69 (C_3_); 25.03 (C_15_); 22.61 (C_8_); 15.37 (C_13_); 14.07 (C_9_); 11.57 (C_14_). ESI-HRMS (*m/z*): Calcd for C_15_H_30_NO_3_^+^ ([M+H]^+^), 272.2215; found, 272.2220.

#### 3.2.11. Methyl 2-(dodecanoylamino)-3-methylpentanoate (*N*-dodecanoyl isoleucine methyl ester)

Using the same reaction and isolation procedure as that for *N*-nonanoyl alanine methyl ester above, *N*-dodecanoyl isoleucine methyl ester was prepared using dodecanoic acid (801.6 mg, 4 mmol), CDI (648.6 mg, 4 mmol), methyl l-isoleucinate (726.6 mg, 4 mmol) in THF (8.79 mL), producing a white solid (1.269 g, 3.85 mmol, 96%). A small portion was purified for characterisation by column chromatography on silica (85% CH_2_Cl_2_/EtOAc, R_f_ = 0.59) producing the title compound as white powder, which was 98% pure (HPLC-MS). ${\left[\alpha \right]}_{D}^{20}$ = +24.1 (*c* 0.76, CHCl_3_). ^1^H-NMR (MeOH-*d*_4_) δ 5.99–5.94 (1H, m, N*H*); 4.62 (1H, dd, *J* = 8.6, 5.0 Hz, H_14_); 2.22 (2H, app t, *J* = 7.6 Hz, H_2_); 1.92–1.84 (1H, m, H15); 1.63 (2H, app q, *J* = 7.3 Hz, H3); 1.44 (1H, dqd, *J* = 14.8, 7.4, 4.7 Hz, H16); 1.36–1.21 (16H, m, H_4-11_); 1.16 (1H, dqd, *J* = 14.8, 7.4, 7.3 Hz, H_13_); 0.92 (3H, t, *J* = 7.4 Hz, H_17_); 0.91 (3H, d, *J* = 6.9 Hz, H_18_); 0.88 (3H, t, *J* = 7.0 Hz, H_9_). ^13^C-NMR (MeOH-*d*_4_) δ 172.91 (C_1_); 172.74 (C_3_); 56.12 (C_14_); 52.07 (C_19_); 37.97 (C_15_); 36.71 (C_2_); 31.87 (C_10_), 30.92, 29.58, 29.46, 29.30, 29.21 (C_4,5,6,7,8,9_); 25.65 (C_3_); 25.18 (C_18_); 22.66 (C_11_); 15.38 (C_16_); 14.10 (C_12_); 11.55 (C_17_). ESI-HRMS (*m/z*): Calcd for C_19_H_38_NO_3_^+^ ([M+H]^+^), 328.2838; found, 328.2846.

#### 3.2.12. 2-(Dodecanoylamino)-3-methylpentanoic acid (*N*-dodecanoyl isoleucine)

Using the same reaction and isolation procedure as that for *N*-nonanoyl alanine above, *N*-dodecanoyl isoleucine was prepared using *N*-dodecanoyl isoleucine methyl ester (1.96 g, 5.98 mmol), THF (4.25 mL), and aqueous NaOH (1 M, 6.1 mL) to produce a white solid. Purification by recrystallisation in 9:1 MeOH/H_2_O overnight at −4 °C produced an organogel, which dissolved at room temperature, and vacuum filtration at 0 °C afforded the title compound (737 mg, 2.35 mmol, 61%) as very fine white crystals that were >98% pure (HPLC-MS). ${\left[\alpha \right]}_{D}^{20}$ = +22.5 (*c* 1.02, CHCl_3_). ^1^H-NMR (MeOH-*d*_4_) δ 6.18–6.09 (1H, m, N*H*); 4.63 (1H, dd, *J* = 8.3, 4.7 Hz, H_14_); 2.22–2.30 (2H, m, H_2_); 1.99–1.92 (1H, m, H_15_); 1.64 (2H, app q, *J* = 7.1 Hz, H_3_); 1.55–1.47 (1H, m, H_16_); 1.36–1.25 (16H, m, H_4-11_), 1.25–1.17 (1H, m, H_16_); 0.96 (3H, d, *J* = 6.8 Hz, H_18_); 0.94 (3H, t, *J* = 7.4 Hz, H_17_); 0.88 (3H, t, *J* = 7.0 Hz, H_12_). ^13^C-NMR (MeOH-*d*_4_) δ 175.48 (C_13_); 173.97 (C_13_); 56.40 (C_14_); 37.59 (C_15_); 36.65 (C_2_); 31.88 (C_10_), 29.58, 29.46, 29.31, 29.29, 29.19 (C_4,5,6,7,8,9_); 25.68 (C_3_); 25.04 (C_18_); 22.67 (C_11_); 15.40 (C_16_); 14.11 (C_12_); 11.58 (C_17_). ESI-HRMS (*m/z*): Calcd for C_18_H_36_NO_3_^+^ ([M+H]^+^), 314.2675; found, 314.2690.

### 3.3. Yeast

Fermentations were carried out with the commercial *Saccharomyces cerevisiae* strain EC1118 (Prise de Mousse, AB Mauri, Australia) with preparation based on the protocol reported by Dennis *et al.* [19].

### 3.4. Fermentation Conditions

#### 3.4.1. Trials Involving Fatty Acids and Precursors

Triplicate fermentations were conducted using model grape juice media (MGJM), prepared as reported previously [19] and sterile filtered prior to use (0.20 μm disposable sterile filter units, Nalgene, Rochester, NY, USA). Controlled fermentations (50 mL) were carried out with individual putative precursor compounds (stock solutions prepared volumetrically using acetone as solvent) spiked into MGJM at 0 μM, 1 μM, 10 μM, 100 μM, and 1 mM and then inoculated with yeast starter culture (1 mL). Air locks were used to maintain an anaerobic environment. Fermentations were conducted in darkness, at ~20 °C, until mass loss stabilised. Yeast cells were removed from the finished fermentations by centrifugation (2300 rcf for 5 min) and the clarified wines were then stored in glass under N_2_ at 4 °C prior to analysis.

#### 3.4.2. Trials Involving Amino Acids and Pantothenate

MGJM was prepared according to the procedure adapted from Henschke and Jiranek [49] and summarised by McBryde *et al.* [50] with some modifications. The medium contained d-glucose (100 g∙L^−1^), d-fructose (100 g∙L^−1^), 3 g∙L^−1^ l-malic acid, 0.2 g∙L^−1^ citric acid, 1.14 g∙L^−1^ K_2_HPO_4_, 1.23 mg∙L^−1^ MgSO_4_**^.^**7H_2_O, 198.2 μg∙L^−1^ MnCl_2_**^.^**4H_2_O, 135.5 μg∙L^−1^ ZnCl_2_, 32 μg∙L^−1^ FeCl_2_, 13.6 μg∙L^−1^ CuCl_2_, 5.7 μg∙L^−1^ boric acid, 29.1 μg∙L^−1^ Co(NO_3_)_2_**^.^**6H_2_O, 24.2 μg∙L^−1^ Na_2_MoO_4_**^.^**2H_2_O, 10.8 μg∙L^−1^ KI, 3.12 g∙L^−1^ KNaC_4_H_4_O_6_**^.^**4H_2_O, 0.44 g∙L^−1^ CaCl_2_**^.^**2H_2_O, 100 mg∙L^−1^ myo-insitol, 2 mg∙L^−1^ pyridoxide HCl, 2 mg∙L^−1^ nicotinic acid, 0.5 mg∙L^−1^ thiamine, 0.2 mg∙L^−1^ *p*-aminobenzoic acid, 125 μg∙L^−1^ biotin, 0.2 mg∙L^−1^ folic acid, 0.2 mg∙L^−1^ riboflavin, and 10 mg∙L^−1^ ergosterol dispersed in 0.5 mL Tween 80 and dissolved in 0.5 mL hot ethanol. The pH of the medium was corrected to 3.4 and sterile filtered (as specified above). The final yeast assimilable nitrogen (YAN) content of each fermentation was adjusted to 450 mg∙L^−1^ by additions of NH_4_Cl solution and target amino acids prepared as follows: NH_4_Cl was dissolved in water at a concentration of 250 g∙L^−1^ and sterilised by filtration; amino acids were prepared as individual stock solutions by dissolving 50 mg of l-cysteine, 320 mg of β-alanine, 500 mg of l-arginine and 200 mg of l-valine in 10 mL sterile water, and 2.5 g of d-pantothenic acid hemicalcium salt was dissolved in 10 mL sterile water.

### 3.5. Quantitative GC-MS Analysis of Esters and Acids

SPME-GC-MS was utilised with the conditions described above under GC-MS instrumentation for the analysis of fermentation treatments. SIM ions are presented in Table 1. A sample of the fermentation medium (1 mL) was diluted volumetrically with sterile water to give a final volume of 10 mL, which was transferred to an SPME vial. NaCl (3 g) was added into each vial along with 10 μL of an ethanolic solution containing d_13_-hexanol (920 mg∙L^−1^), *d*_11_-hexanoic acid (930 mg∙L^−1^), *d*_5_-ethyl nonanoate (9.2 mg∙L^−1^), *d*_16_-octanal (82.1 mg∙L^−1^) and *d*_3_-linalool (1.73 mg∙L^−1^), and 10 μL of an ethanolic solution containing d_3_-hexyl acetate (8.76 mg∙L^−1^) prior to analysis. For quantifying the analytes, triplicate standards in model wine (12% aqueous ethanol, pH adjusted to 3.2 with tartaric acid) were prepared at evenly spaced concentrations across the range. The highest standard concentration for each analyte was approximately 150% of the highest concentration observed in the model wines. Calibrations were linear throughout the range with R^2^ = 0.97–0.99. All calibration samples were prepared and analysed according to the protocols outlined above.

### 3.6. Determination of β-Alanine Content of Grape Juice

β-Alanine was quantified in grape juice using an AccQ Fluor Reagent Kit (Waters Corporation, Milford, MA, USA) and a method based on that of Cohen and Michaud [51]. An SPE step was added prior to derivatisation to reduce chromatographic background [52]. The grape juice was obtained from a 200 mg aliquot of frozen ground grape material that had been prepared from approximately 25 g of berries. The samples were all from the Cabernet Sauvignon variety and were obtained from commercial vineyards in the Barossa Valley, Clare Valley, Coonawarra, Eden Valley, Langhorne Creek, McLaren Vale, Riverland and Wrattonbully grapegrowing regions of South Australia in the 2013 and 2014 vintages.

**Table 1:** Qualifier and quantifier ions used for GC-MS analysis of fermentation volatiles in SIM mode.

Analyte (Internal Standard)	SIM Ions (Quantifier in Bold)
Ethyl acetate	43, 61, **70**
Ethyl hexanoate	43, 60, **88**
Ethyl octanoate	**88**, 101, 127
Ethyl nonanoate	73, **88**, 101, 141, 186
Ethyl decanoate	73, **88**, 101, 157
Ethyl dodecanoate	43, **88**, 101, 157, 183
Isoamyl acetate	43, 55, **70**, 87
Phenyl ethyl acetate	39, 43, 65, 91, **104**
Hexanoic acid	41, **60**, 73, 87
Octanoic acid	43, 55, **60**, 73, 101
Decanoic acid	57, **60**, 73, 101, 129
(d_3_-Hexyl Acetate)	46, 59, **84**
(d_5_-Ethyl Nonanoate)	41, 60, **93**
(d_11_-Hexanoic Acid)	**63**, 71

### 3.7. Data Analysis

Results are presented as the mean of triplicate fermentation samples. The results were analysed by one-way analysis of variance (ANOVA) followed by the Games-Howell (fatty acid experiments) or Tukey’s (amino acid experiments) *post hoc* tests using SPSS version 20 (IBM; Armonk, NY, USA).

## 4. Conclusions

Understanding the links between grape composition and concentrations of volatile compounds in wine has important implications with respect to viticultural and winemaking parameters and the impact of these on wine style and quality. Based on previous studies and an examination of yeast biochemical pathways, known and putative grape-derived precursors were assessed for their ability to produce ethyl and acetate esters upon fermentation in chemically defined model media. Significant stimulating effects of MCFAs and their derivatives were revealed, as was the apparent toxicity of some compounds at the highest concentrations tested. MCFAs, their methyl esters and carnitine conjugates were able to contribute to ethyl ester production, albeit with variable influences of odd and even chain length MCFAs. Amino acid conjugates of MCFAs also produced the corresponding ethyl esters, but with differences evident between C_9_ and C_12_ MCFAs and for alanine compared to isoleucine; a toxic effect was noted at higher concentrations of nonanoyl alanine, whereas dodecanoyl isoleucine had no significant effect on ester production.

Investigation of four amino acids related to CoA production by yeast revealed the exclusive effect of β-alanine on the formation of a range of MFCAs and their ethyl esters, along with acetate esters. Ethyl hexanoate, octanoate and decanoate significantly increased with 50 and 100 mg∙L^−1^ of β-alanine, as did their MCFA counterparts, along with ethyl and phenylethyl acetates, but not isoamyl acetate. The results implied a stimulatory effect on MCFA synthesis, leading to corresponding increases in esters, but isoamyl acetate levels were deemed to be limited by isoamyl alcohol concentrations. Additional experiments with lower concentrations of β-alanine, and β-alanine along with pantothenate, showed that levels at or below the usual grape concentrations of these compounds were able to stimulate production of a range of MCFAs and their ethyl esters, as well as ethyl, isoamyl and phenylethyl acetate esters. Both β-alanine and panthothenate promoted ester production but β-alanine was more effective in some circumstances.

Overall, a range of putative and known grape-derived precursors were shown to have important effects on the production by yeast of MCFAs, and ethyl and acetate esters. These interesting outcomes open the way for additional investigations of odd *versus* even MCFA chain length, MCFA carnitine derivatives and the importance of β-alanine relative to pantothenate in actual juices or musts, which may reveal even greater insights into the relationship between nonvolatile grape components and wine aroma constituents. Ultimately, being able to predict and control wine aroma based on grape composition and winemaking inputs could be realised as a result of such studies.
